# Pancreatic duplication cyst misdiagnosed as distal pancreatic tumor: A case report and surgical approach

**DOI:** 10.3389/fsurg.2023.1148308

**Published:** 2023-03-10

**Authors:** Lara Zain, Raghad Sweity, Khaled Alshawwa, Sami Bannoura, Bashar Jaber, Omar Abu-Zaydeh

**Affiliations:** ^1^ Medical Research Club, Faculty of Medicine, Al-Quds University, Jerusalem, Palestine; ^2^ Department of General Surgery, Al-Makassed Charitable Society Hospital, Jerusalem, Palestine; ^3^ Department of Pathology, Al-Makassed Islamic Charitable Society Hospital, Jerusalem, Palestine

**Keywords:** pancreas, pancreatic neoplasms, pancreatic duplication cyst, gastric-type duplication cyst, pancreatic pseudocysts, surgical resection.

## Abstract

Enteric duplication cysts (EDCs) are a benign and uncommon congenital malformation, with a nonspecific and extremely variable clinical presentation. EDCs associated with the pancreas are called pancreatic duplication cysts (PDCs). They are especially rare and can present with recurrent abdominal pain or even severe pancreatitis. These cysts often get confused with pancreatic neoplasms or pseudocysts, thus posing diagnostic and surgical challenges. Here, we report a case of a 20-year-old male patient with a 14-year history of recurrent abdominal pain and many hospital admissions, who had several imaging studies revealing a persistent focal heterogeneous lesion affecting the tail of the pancreas, surrounding a small pseudocyst. An ultrasound (U/S) guided biopsy was avoided due to the location of the mass. Surgical resection was carried out for the suspicion of malignancy and final pathology report showed benign findings while revealing that what was thought to be a pseudocyst turned out to be a gastric-type PDC, and after reviewing the available literature, we encountered 16 similar cases regarding misdiagnosing PDCs. We conclude that PDCs are very rare and have a variable clinical presentation as well as a likelihood of being confused with other pancreatic neoplasms. Therefore, PDCs need a high index of suspicion to avoid recurrent hospital admissions and unnecessary procedures due to the fact that sometimes a simple cystectomy is adequate.

## Introduction

Enteric duplication cysts (EDCs) are rare and very uncommon congenital malformations with a reported incidence of 1 in every 4,500 live births (1). In order to classify a cyst into being an EDC, it needs to have a well-developed smooth muscle coat, a mucosal lining of ectopic cells found within some portion of the digestive system, and some contiguity with any part of it (2). The origin of EDCS is believed to be multifactorial, as several theories have been proposed in an attempt to reach a specific etiology, but no one had the ability to justify the exact cause since gastrointestinal duplication cysts can occur anywhere along the alimentary tract, with a percentage being completely isolated and some being associated with different anomalies (3).

An EDC is named based on its anatomical location, as it generally shares the same smooth muscle and blood supply of the adjacent organ (2), but may or may not be connected to its lumen. However, its location doesn't predict the ectopic cells it contains (4).

Most EDCs are detected within the first two years of life and scarcely in adulthood. The most common ones are ileal duplication cysts and the rarest being the isolated EDCs, which have their own blood supply and muscular wall (5).

EDCs that are present in the pancreas are called Pancreatic duplication cysts (PDC), and are considered a very rare entity (6) that may appear in the body, head, or tail of the pancreas. They are difficult to explain due to lack of communication with the alimentary tract, shared pancreatic blood supply, and the possibility of being connected to the pancreatic duct; as one theory proposes that inflammation and ulceration within the cyst result in perforation into the duct of the adjacent pancreas (6). Clinical presentation varies from being completely asymptomatic to having recurrent episodes of abdominal pain or even pancreatitis. Their diagnosis presents a challenge; as they are often misdiagnosed with pseudocysts or other pancreatic tumors, leading to incorrect management. But once diagnosed, surgical excision is the cornerstone of therapy whether symptomatic or incidental; due to the possibility of malignant transformation (7). Therefore, diagnosing PDCs allows for appropriate surgical intervention with simple cystectomy being an adequate choice unless there is extensive pancreatic scarring or malignancy requiring radical resections (8).

Here, we report a case of a 20-year-old male patient with a history of recurrent abdominal pain, who had several imaging studies revealing a persistent lesion affecting the tail of the pancreas. Surgical resection was carried out and the final pathology report revealed benign findings with a gastric-type PDC.

## Case description

A 20-year-old male patient presented to our hospital in February 2022, for further evaluation of recurrent attacks of abdominal pain of 14-year duration. He had free past medical and surgical histories.

The patient was 6 years old when he started to complain of severe dull aching pain in the mid upper abdomen that radiated to the back and was associated with vomiting of normal gastric content. The attacks happened at least 4 times per year. During each attack, an abdominal ultrasound showed unremarkable findings, and the patient was only treated with intravenous analgesics.

In 2018, the pain became intolerable as it increased in severity and frequency. An abdomino-pelvic computed tomography (CT) scan was done and revealed splenic vein thrombosis with a Pancreatic Pseudocyst. The patient was treated with anticoagulation, and a follow-up U/S showed the resolution of the thrombus. However, the patient was still complaining of the same attacks of abdominal pain, for which he underwent Magnetic resonance cholangiopancreatography (MRCP) that showed a small pseudocyst formation measuring about 0.8 cm and surrounded by heterogeneous pancreatic tissue measuring about 2.3 cm with features of inflammatory changes; which led to the suspicion of malignancy. Therefore, surgery was advised, but the patient refused and continued to have the same attacks with no improvement.

In January 2022, the patient underwent a CT scan, which revealed a persistent focal heterogeneous mass like lesion containing a small pseudocyst affecting the pancreatic tail. Then in February 2022, an abdominal ultrasound showed a hypovascularized, heterogeneous mass at the most distal portion of the pancreatic tail. Therefore, he was admitted to our hospital on February 27, 2022 as a case of a suspicious pancreatic tail mass. The physical examination was unremarkable, and the last episode of abdominal pain was one week prior to admission.

Serum lipase and amylase levels were within the normal range and Carcinoma embryonic antigen (CEA) and Cancer antigen 19-9 (CA19-9) were 1.8 ng/dl and 8.1 U/ml respectively. U/S guided endoscopic biopsy was disfavored due to splenic flexure interposition and a risk of colon perforation or splenic hemorrhage.

**Figure 1:**
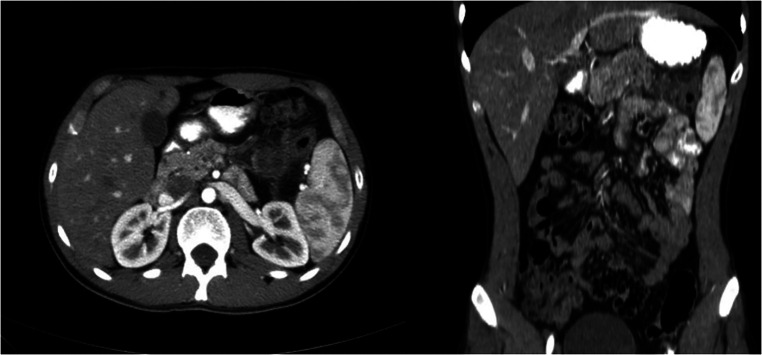
CT scan (axial and coronal) cuts showing lobulated hypo-dense lesion in the pancreatic tail, measuring 3 cm × 2.5 cm with minimal rim enhancement contains at least three cysts, the largest measuring 1.5 cm with stranding of the adjacent mesenteric fat.

The patient remained stable with no complaints and a laparoscopic distal pancreatectomy was planned for March 06, 2022. A CT-Scan Pancreas Protocol (three phases) showed a lobulated hypodense lesion in the tail of the pancreas, measuring 3 cm × 2.5 cm with minimal rim enhancement that contains at least three cysts; the largest measuring 1.5 cm with stranding of the adjacent mesenteric fat (Figure 1).

Due to the suspicion of malignancy and to look for other primary malignancies; a gastroscopy was done which showed mild superficial antral gastritis and a colonoscopy only reached the splenic flexure, with no further progression either due to severe angulation or extra colonic compression. Then, the patient underwent surgery that consisted of distal pancreatectomy, splenectomy and segmental resection of the colonic splenic flexure that were sent to the histopathology lab.

The surgery started laparoscopically by dissecting around the splenic flexure and the inferior and superior pancreatic borders, but due to the mass adherence to the colon, and the need for colonic resection and anastomosis it was difficult to continue laparoscopically. So, through a reverse Kocher incision, the abdomen was opened.

During the procedure, the lesion was found to be proximal to the spleen, but a splenic-preserving pancreatectomy was not possible due to the inability to isolate and protect both of the splenic artery and vein, thus necessitating the removal of the spleen.

On postoperative day 7, the patient was ready to be discharged as he was stable and tolerating a regular diet, with the right drain being kept in place.

On follow-up (December 2022) the patient was doing well, he got all of the indicated vaccines two weeks after discharge that included: pneumococcal, meningococcal and Haemophilus influenzae vaccinations. In addition, he only experienced one attack of abdominal pain two months after the surgery; it was mild, did not require hospitalization and was relieved with non-steroidal anti-inflammatory drugs. After that, he did not experience any abdominal pain.

Final pathology report demonstrated a 0.8 cm cyst near the tail of pancreas with a smooth muscle wall and gastric mucosa lining consistent with a gastric-type pancreatic duplication cyst and no evidence of malignancy, as the suspected mass consisted of inflammatory tissue with hemosiderin deposition and fibrosis. (Main findings and procedures are summarized in Table 1).

**Table 1:** Timeline table with main findings and procedures.

Date	Investigations and/or Procedures
2018	Abdomino-pelvic CT scan: Splenic vein thrombosis and pancreatic Pseudocyst
September 2018	MRI of the Abdomen & MRCP: Focal cystic area in the tail of pancreas, focal prominent pancreatic duct in tail, small cyst 0.8 cm, mild surrounding fat edema, focal heterogeneous pancreatic tissue 2.3 cm and features of inflammatory changes.
January 2022	Abdomino-pelvic CT: focal heterogeneous enhancing mass like lesion measured about 2.8 cm × 3.5 cm, affecting the tail of pancreas with a focal prominent pancreatic duct with small pseudocyst measured about 8 mm, surrounded by fat stranding.
February 2022	Abdominal U/S: heterogeneous hypovascularized mass at the most distal portion of the pancreatic tail measuring about ∼3.3 cm × 2.1 cm.
March 04, 2022	CT-Scan Pancreas Protocol (3 phaser): a lobulated hypodense lesion in the pancreatic tail measuring 3 cm × 2.5 cm with minimal rim enhancement contains at least three cysts, the largest measuring 1.5 cm with stranding of the adjacent mesenteric fat.
March 06, 2022	Surgical intervention that consisted of distal pancreatectomy, splenectomy and segmental resection of colonic splenic flexure
March 13, 2022	Histopathology report: • Negative for malignancy • A mass consisting of inflammatory tissue with hemosiderin deposition and fibrosis. • A 0.8 cm duplication cyst identified near the tail of pancreas consisting of gastric mucosa.

## Diagnostic assessment

The presentation of our patient with recurrent abdominal pain, several hospital admissions and imaging studies revealing suspicious features pointed towards a malignant process. Pseudocysts are not typically operated but the surrounding hypodense and heterogeneous tissue obligated a surgical approach for this case. A distal pancreatectomy, splenectomy and segmental resection of the splenic flexure were carried out, and the specimens were sent for histopathology that thankfully came back benign, but to our surprise; what was thought to be a pseudocyst turned out to be a pancreatic EDC with a gastric-type mucosa, which was the underlying pathology that caused our patient to suffer for 14 years.

## Discussion

PDCs are very rare with a non-specific presentation. There have been 16 cases encountered in the literature (6, 9–21) reporting misdiagnosing PDCs as pancreatic neoplasms (Table 2). Most patients were asymptomatic and the cysts were found incidentally (6, 10, 11, 15, 17, 19, 21), while the rest came with recurrent symptoms (6, 11, 12, 14, 18, 20). Symptomatic cases of PDCs presented with recurrent abdominal pain and sometimes pancreatitis, owing to inflammation and compression of the pancreatic duct leading to elevated serum amylase and lipase levels (1). In these cases, as well as in our patient, the diagnosis was almost always delayed for several years, with patients getting supportive treatment and being sent back home and then coming back again with a similar episode. However, more severe presentations, such as GI bleeding and even perforation have been encountered. All of which depends on the size of the cyst and its mucosal lining (1), which might have contributed to some cases presenting acutely (9, 13, 16) and others requiring urgent surgical intervention, especially the ones associated with ectopic gastric or pancreatic mucosa (1).

**Table 2:** Summary of encountered case reports with misdiagnosed pancreatic enteric duplication cysts.

gender	Age at diagnosis	Presentation	Fine needle aspiration	References
F	22	Presented acutely with abdominal pain, nausea and vomiting (n/v)	Presence of Mucin	(9)
F	52	Incidentally found	High amylase, CEA and CA 19-9	(10)
M	24	3-to 4- weeks of Abdominal pain, lethargy, loose stool and nausea	High CEA	(11)
F	49	Incidentally found	High CEA and CA 19-9	(11)
F	43	1-year history of chronic Abdominal pain	High CEA and amylase	(12)
F	44	3-day history of right flank and lower quadrant abdominal pain with constipation and nausea	Mucin and high CEA	(13)
M	38	Recurrent episodes of Abdominal pain	High amylase and CEA	(14)
F	45	Incidentally found	–	(15)
F	7	Increasingly severe abdominal pain after milder symptoms for a week.	High amylase, lipase, CEA and CA 19-9	(16)
F	46	Incidentally found	–	(17)
F	48	4-year history of intermittent abdominal pain with n/v	High CEA and amylase	(18)
F	63	Incidentally found	–	(19)
M	35	Incidentally found	High amylase	(6)
F	28	Repeated episodes of epigastric and right upper quadrant pain with n/v since the age of 8 years.	–	(6)
F	57	12-month history of recurrent severe upper abdominal pain radiating to the back associated with occasional episodes of vomiting.	High CA 19-9 and CEA	(20)
M	71	Incidentally found	–	(21)

The diagnosis of a PDC presents a dilemma and it may be confused with other pancreatic lesions that may delay the diagnosis and put the patient through inappropriate procedures. Moreover, by using different imaging modalities such as endoscopic ultrasound (EUS), MRI or a CT scan, it's not guaranteed to identify the cystic nature of a PDC. Even though some modalities are superior to others like the EUS, the thick proteinaceous cyst fluid makes it difficult to assess (10). Therefore, on imaging, a mass will be demonstrated, for which the differential diagnoses will still include all the pancreatic lesions such as pseudocysts, cystic neoplasms and PDCs (22).

It's worth mentioning that a CT scan can provide helpful information about the precise anatomical relationship between the cysts and surrounding structures, making it easier to distinguish duplications arising from the stomach, duodenum, and pancreas (23). Also, when a CT scan is done with intravenous contrast, it can sometimes demonstrate the enhancement of the enteric mucosa within the PDC itself (24). Additionally, an EUS helps in identifying something called the “gut signature” of a PDC which is the serosa appearing as an outer hypoechoic layer and the mucosa appearing as an inner hyperechoic layer, and it may also demonstrate peristalsis of the cyst's wall that is strongly suggestive of a duplication cyst (25).

Despite this, a 50% incidence of misdiagnosis still remains (26), often leading to inappropriate operations and management. In our patient, imaging modalities played an indispensable role, they were able to provide valuable information about the location and characteristics of the mass, but they didn't show any clue to help identify it as a PDC. Instead, showing a hypodense, heterogenous, lobulated mass with adjacent organ involvement led to the suspicion of a neoplastic process.

As a part of evaluating pancreatic cystic lesions, fine needle aspiration is used to determine malignant potential, but this contributed to some cases of PDCs being misdiagnosed as pancreatic neoplasms (13–18, 20, 22, 25), and that's because fluid analysis showed either the presence of mucin mimicking Mucinous cystic pancreatic neoplasms, or high CEA and CA19-9 tumor markers mimicking other neoplastic processes. In our patient, it was avoided due to the location of the suspected mass which made it technically difficult to perform.

**Figure 2:**
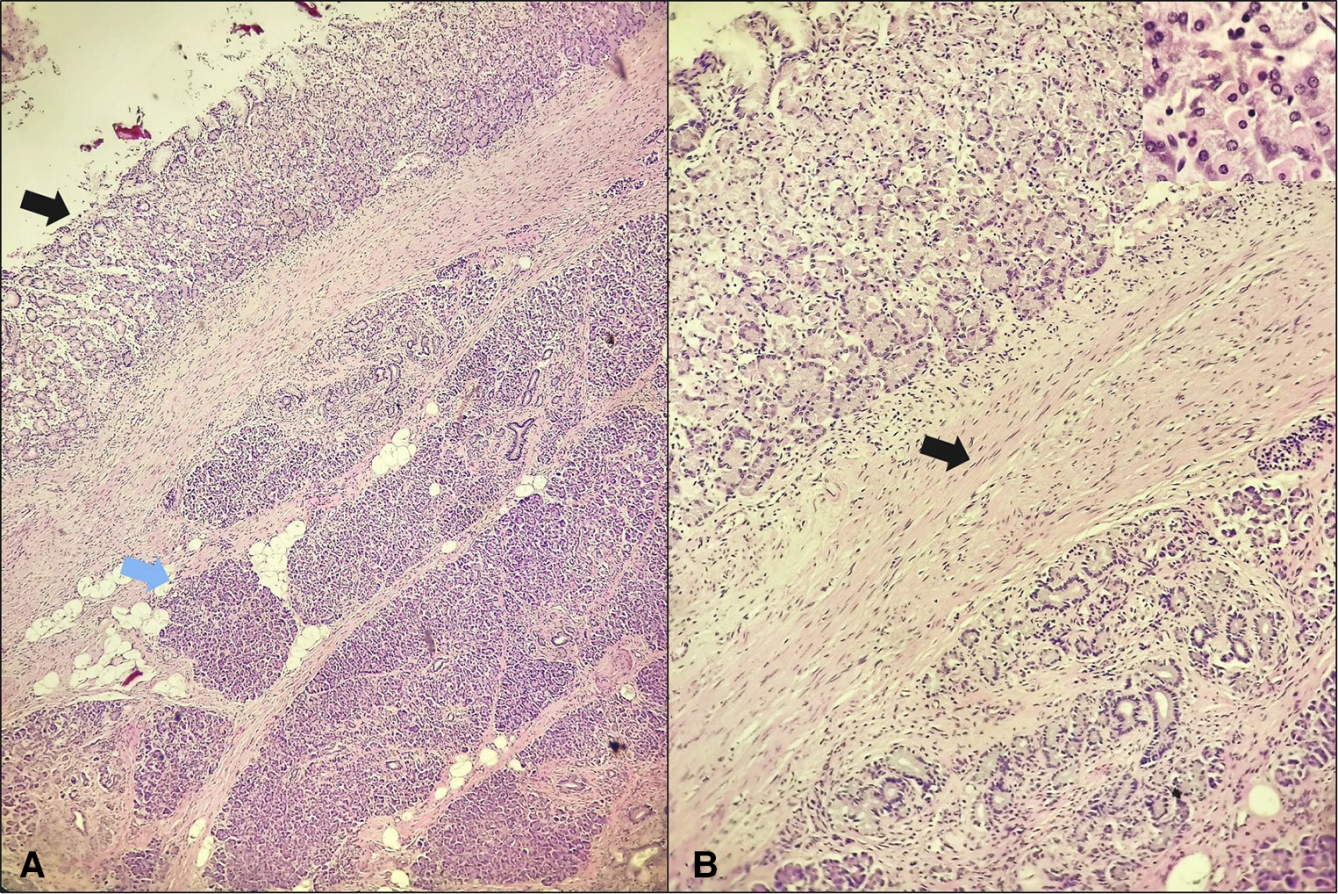
Gastric-type duplication cyst in the pancreas. (**A**) The cyst lining appears in the left upper corner (black arrow) and is separated from the underlying pancreatic parenchyma (blue arrow) by a fibrous connective tissue with presence of smooth muscle (H&E; 4×); (**B**) the cyst lining is consistent with corpus-type gastric mucosa with both chief and parietal cells seen (insert), the cyst wall contains smooth muscle (arrow) (H&E; 10×, insert 40×).

Therefore, most cases of PDCs are diagnosed after surgical resection by histopathologic examination of the surgical specimen, which was the case in our patient. Classically on histologic examination, a duplication cyst wall will show layers of smooth muscle mimicking those of the adjacent gut wall; which is demonstrated in our specimen (Figure 2). And the cyst's lining will show any type of mucosa found within the digestive system; in our case, it was consistent with corpus-type gastric mucosa with both chief and parietal cells (Figure 2B). Although, there has been a case reported where the mucosa had been partially denuded and the muscular coat was found to be fibrotic owing to extensive chronic inflammation (27). Hence, in such cases distinguishing a PDC from a pseudocyst on histopathology can be really difficult.

But once a PDC is diagnosed, surgical resection is the mainstay of therapy, and the approach depends upon the pathology encountered. If the separation of the cyst from the surrounding structures is possible, simple cystectomy is sufficient (8). In our case, Imaging findings, preoperative investigations and the evidence of adjacent organ involvement all failed to exclude malignancy; which obligated a distal pancreatectomy, splenectomy and segmental resection of the colonic splenic flexure.

To conclude, PDCs are difficult to differentiate from other tumors of the pancreas unless some clues appear on imaging studies. Thus, they might remain misdiagnosed until they are surgically resected. Recurrent abdominal symptoms or events such as bleeding, perforation and the reported possibility of a malignant transformation point towards having surgical resection as the first treatment option for PDCs.

## Data Availability

The original contributions presented in the study are included in the article/Supplementary Material, further inquiries can be directed to the corresponding author.
